# Retraction: DDP-resistant ovarian cancer cells-derived exosomal microRNA-30a-5p reduces the resistance of ovarian cancer cells to DDP

**DOI:** 10.1098/rsob.210329

**Published:** 2021-11-17

**Authors:** Ronghua Liu, Yucan Zhang, Peiwen Sun, Changxiu Wang

*Open Biol.* **10**, 190173. (Published 29 April 2020). (doi:10.1098/rsob.190173)

The Editor-in-Chief in agreement with the Publisher has retracted this article. Following an investigation, several flaws and inconsistencies in the flow-cytometry cell-cycle analyses and western blots were found. The authors have not been able to supply the raw data as requested, thus, the editors consider the conclusions of this article to be invalid. The authors have not responded to correspondence about this retraction.

The image integrity standards and policies for the journal can be found here: https://royalsocietypublishing.org/doi/10.1098/rsob.200165.

Jonathon Pines FRS, Editor-in-Chief, *Open Biology*.

